# P-1007. Cytomegalovirus reinfections and viral shedding in seropositive pregnant women

**DOI:** 10.1093/ofid/ofae631.1197

**Published:** 2025-01-29

**Authors:** Shannon Ross, Suresh Boppana, Anslee Vinson, Karen Fowler

**Affiliations:** University of Alabama at Birmingham, Birmingham, Alabama; University of Alabama at Birmingham, Birmingham, Alabama; University of Alabama at Birmingham, Birmingham, Alabama

## Abstract

**Background:**

Congenital Cytomegalovirus (CMV) is a common cause of hearing loss and other sequelae in children. Rates of congenital CMV are higher in maternal populations seropositive before pregnancy, often due to the acquisition of a new strain of CMV. The objective of this study is to document CMV reinfections in CMV seropositive pregnant women and correlate them with CMV shedding.

**Methods:**

Pregnant women positive for CMV IgG were enrolled after their first prenatal visit but before 20 weeks gestation and followed prospectively. Maternal demographics were obtained, and blood, urine, saliva, and vaginal samples were obtained at enrollment and regular intervals during pregnancy and immediately post-partum. Maternal sera were analyzed for strain-specific anti-CMV antibody reactivity to four CMV epitopes, and when a new strain-specific antibody reactivity occurred during pregnancy, they were considered reinfected. All specimens were tested for CMV DNA by Real-time PCR.

**Results:**

Among 165 pregnant women, the mean age at enrollment was 26.6 years (±5.4). 80% of the women were non-Hispanic Black and never married. At study enrollment, 52 women (31.5%) had antibody reactivity to one CMV strain while 83 (50%) had 2 or more strain-specific antibodies. There was no difference in viral shedding at study enrollment between the women with one strain-specific antibody (4/52, 7.7%) compared with women with two or morestrain specific antibodies (11/83, 13.3%, p=0.34). Ten of 165 women (6%) acquired antibodies reactive with a new strain of CMV during the study period (reinfection). Among the 10 women with CMV reinfection, 3 (33.3%) had CMV shedding, with 2 shedding in the visit before documented serological reinfection and one with viral shedding after reinfection. The majority of women with reinfection provided direct care of young children (9/10), lived in a household with 3 or more people (8/10), and lived with children < 6 years of age (8/10).

**Conclusion:**

In this urban, young cohort of seropositive pregnant women, CMV reinfections occur. However, viral shedding was documented in only a third of women with new CMV antibody reactivity. Most women with reinfection had close contact with young children.

**Disclosures:**

**Suresh Boppana, MD**, GSK: Advisor/Consultant|Merck: Grant/Research Support|Pfizer: Grant/Research Support **Karen Fowler, DrPH**, Moderna: Advisor/Consultant

